# Advances in Shotgun Metagenomics for Cheese Microbiology: From Microbial Dynamics to Functional Insights

**DOI:** 10.3390/foods15020259

**Published:** 2026-01-10

**Authors:** Natalia Tsouggou, Evagelina Korozi, Violeta Pemaj, Eleftherios H. Drosinos, John Kapolos, Marina Papadelli, Panagiotis N. Skandamis, Konstantinos Papadimitriou

**Affiliations:** 1 Department of Food Science and Technology, University of the Peloponnese, 24100 Antikalamos, Greece; n.tsouggou@go.uop.gr (N.T.); v.pemaj@go.uop.gr (V.P.); i.kapolos@go.uop.gr (J.K.); m.papadelli@go.uop.gr (M.P.); 2 Laboratory of Food Quality Control and Hygiene, Department of Food Science and Human Nutrition, Agricultural University of Athens, Iera Odos 75, 11855 Athens, Greece; evangeliakorozi95@gmail.com (E.K.); ehd@aua.gr (E.H.D.); pskan@aua.gr (P.N.S.)

**Keywords:** shotgun metagenomics, microbial ecology, MAGs, antimicrobial resistance, ARGs, bacteriocins, functional analysis, CRISPR, virulence, phage, cheese, microbiome

## Abstract

The cheese microbiome is a complex ecosystem strongly influenced by both technological practices and the processing environment. Moving beyond traditional cultured-based methods, the integration of shotgun metagenomics into cheese microbiology has enabled in-depth resolution of microbial communities at the species and strain levels. The aim of the present study was to review recent applications of shotgun metagenomics in cheese research, underscoring its role in tracking microbial dynamics during production and in discovering genes of technological importance. In addition, the review highlights how shotgun metagenomics enables the identification of key metabolic pathways, including amino acid catabolism, lipid metabolism, and citrate degradation, among others, which are central to flavor formation and ripening. Results of the discussed literature demonstrate how microbial composition, functional traits, and overall quality of cheese are determined by factors such as raw materials, the cheesemaking environment, and artisanal practices. Moreover, it highlights the analytical potentials of shotgun metagenomics, including metagenome-assembled genomes (MAGs) reconstruction, characterization of various genes contributing to flavor-related biosynthetic pathways, bacteriocin production, antimicrobial resistance, and virulence, as well as the identification of phages and CRISPR-Cas systems. These insights obtained are crucial for ensuring product’s authenticity, enabling traceability, and improving the assessment of safety and quality. Despite shotgun metagenomics’ advantages, there are still analytical restrictions concerning data handling and interpretation, which need to be addressed by importing standardization steps and moving towards integrating multi-omics approaches. Such strategies will lead to more accurate and reproducible results across studies and improved resolution of active ecosystems. Ultimately, shotgun metagenomics has shifted the field from descriptive surveys to a more detailed understanding of the underlying mechanisms shaping the overall quality and safety of cheese, thus bringing innovation in modern dairy microbiology.

## 1. Introduction

Cheese represents a dynamic microbial ecosystem in which microorganisms influence fermentation, ripening, flavor development, and product safety. Most often, this ecosystem arises from a complex succession of microbial populations that are either intentionally introduced at the beginning of production (e.g., starter cultures) or adventitiously acquired from the raw materials and the surrounding environment. The dynamic equilibrium among bacteria, yeasts, and molds involves complex ecological relationships such as competition, commensalism, and syntrophy, which collectively drive the biochemical transformations occurring during ripening. These microbial interactions drive diverse metabolic processes that ultimately shape the properties of the final product. Therefore, understanding in detail the cheese microbiome is crucial for optimizing the production process and for preserving and improving desirable quality and safety attributes. According to the modern conceptual framework, the microbiome encompasses not only the community of microorganisms (*microbiota*) present in each habitat, but also their collective genomes, metabolites, and the surrounding environmental conditions that influence their activity. This holistic definition reflects the view of the microbiome as an integrated ecosystem, rather than merely a collection of taxa [1,2].

Historically, the investigation of cheese microbiota relied on culture-based microbiological techniques, which provided valuable insights into the culturable microbial fraction, but could not describe the proportion of unculturable taxa [3]. Subsequently, molecular methods such as PCR-based assays, Sanger-based 16S rRNA gene sequencing, denaturing gradient gel electrophoresis (DGGE)/temperature gradient gel electrophoresis (TGGE), and qPCR contributed to the identification of microorganisms independently of their culturability, at least to a degree [4]. The introduction of the next-generation sequencing (NGS) technologies employed for 16S rRNA gene sequencing and internal transcribed spacer (ITS) amplicon sequencing, broadened the field by enabling high-throughput culture-independent profiling of microbial communities. However, despite these advances, amplicon-based approaches are restricted by PCR bias and limited taxonomic resolution, typically reaching up to the genus level [5,6].

These limitations paved the way for the development and integration of shotgun metagenomics in cheese studies, which allows comprehensive analysis of the total microbial ecosystem present in a sample. Shotgun metagenomics relies on the direct extraction and sequencing of total community DNA from environmental samples without prior knowledge of their microbial composition [3,7].

When combined with advanced bioinformatics, it allows the detection of intraspecies genetic variations, metabolic pathway reconstruction, and the identification of various gene clusters (e.g., flavor formation related genes, antimicrobial resistance genes, etc.). Such datasets therefore provide detailed taxonomic and functional insights into complex microbial ecosystems and permit an in-depth understanding of microbial adaptation and evolution across diverse environments [8,9]. In silico reconstruction of metagenome-assembled genomes (MAGs) can reveal technologically important features of taxa [10,11,12].

The reduction in sequencing costs, together with the expansion of computational capacity, have established shotgun metagenomics as a crucial tool in food microbiology research. Continuous improvements and optimization of wet-lab techniques along with the refinement of downstream analyses tools have led to the development of new workflows tailored to dairy science. Multiple bioinformatic engines now enable taxonomic profiling and functional characterization of complex communities with high accuracy, supporting both raw read-based and assembly-based analyses and allowing meaningful conclusions to be drawn at multiple stages of metagenomic workflows. Shotgun metagenomics combined with other “omics” techniques such as metabolomics, volatilomics, and proteomics, which provide additional layers of complexity, can link the microbiome to specific biochemical processes, expanding our comprehension of microbially driven technological properties of dairy products [3]. For example, the combination of shotgun metagenomics with volatilomics to study flavor development in Buffalo Mozzarella and surface-ripened cheeses has linked several microorganisms to distinct groups of volatile compounds [13,14].

This study reviews recent applications of shotgun metagenomics in cheese research, highlighting how this approach has advanced the characterization of cheese microbiomes beyond traditional culture-based and amplicon-based methods. It integrates studies that resolve microorganisms at species/strain level and link their functional potential to cheese quality and safety. By consolidating this rapidly expanding but fragmented body of literature, this review provides a mechanistic understanding of microbiome-driven cheese properties derived from shotgun metagenomics.

## 2. The Literature Search Strategy

Relevant studies were identified by searching PubMed and Scopus using the keywords “shotgun metagenomics cheese”, “shotgun metagenomic cheese”, “shotgun metagenome cheese”, and “metagenomics cheese”. No explicit time-frame restrictions were applied in order to capture all available studies published following the establishment and widespread adoption of NGS technologies. Research articles reporting the application of shotgun metagenomics approaches to cheese were considered eligible and included in the review.

## 3. Wet-Lab Design Considerations in Cheese Shotgun Metagenomics

Appropriate sample handling prior to NGS and downstream bioinformatic analysis is a critical step for ensuring the accuracy and reliability of shotgun metagenomics data. Key methodological challenges reported in the literature, together with commonly applied mitigation strategies, are summarized in Table 1, which highlights important considerations for the design of cheese-focused metagenomics studies.

DNA extraction from dairy products may be hindered by the presence of fats, proteins, and calcium [15]. In addition, host DNA may interfere with microbial signals during NGS, leading to low-quality reads, reduced coverage and fragmented assemblies. To overcome these, various protocols have been developed, and host-depletion kits have also been successfully applied, resulting in improved microbial DNA recovery, taxonomic profiling, and MAG reconstruction [16].

The inability to distinguish DNA from viable versus dead cells is another limitation that may cause bias [17,18]. During ripening, certain microorganisms die or enter a viable but non-culturable (VBNC) state due to the multiple stress conditions established within the cheese. Their DNA may be trapped within the inactivated cells or still be present in the cheese matrix. Propidium monoazide (PMA) treatment could be used to address this issue. In a study conducted on aged Cheddar cheese, this approach enabled the clear detection of the decline of *Lactococcus cremoris* as ripening progressed, in contrast with the gradual increase in *Lacticaseibacillus paracasei* [19]. PMA combined with metagenomics analyses can help determine the metabolically active or inactive members. Of note, additional approaches to discriminate between live and dead cells such as the application of ethidium monoazide (EMA) or DNase pre-treatment to remove extracellular DNA have been employed in other biological matrices but they have not been reported in shotgun metagenomic studies of cheese yet. Sampling methodology is another major factor influencing scientific outcomes. Cheese metagenomes can be highly heterogeneous, since the different parts such as the core and rind, as well as cheese parts exposed to the environment or in contact with the processing surfaces, may be uniquely shaped due to the conditions of each distinct part. For example, the rinds of surface- or cave-ripened cheeses were found to contain remarkably distinct microbial compositions from the respective cores of the same cheeses [13,20,21]. Another challenge in metagenomics studies is the accuracy of quantification, which is typically expressed as relative abundance [22]. This approach can introduce comparison biases, as the extracted microbial loads may vary significantly across samples, matrices, or time points. However, integrating experimental normalization steps such as spike-in controls, or quantifying microbial populations using flow cytometry or qPCR can provide more accurate cell count and ensure reliable comparisons. Constant improvement of experimental designs and data handling by integrating such strategic steps in cheese research will further strengthen the power of cheese shotgun metagenomics.

**Table 1:** Wet-lab challenges and mitigation practices.

Wet-Lab Bias	Impact on Cheese Metagenome	Suggested Mitigation Practices	References
DNA extraction difficulties due to high protein and fat content	Decrease in DNA yield leading to underrepresentation or overrepresentation of certain microorganisms with a concurrent bias in relative abundance.	Application of experimental protocols adjusted for dairy matrices including protein and fat removal steps, as well as mechanical and enzymatic cell lysis. Different kits evaluation on test samples.	[15,23,24,25]
Host DNA interference	Host DNA (e.g., bovine, ovine, caprine) can appear in high levels in the extracted DNA, thus interfering with the coverage of microbial reads, especially those found in low abundance.	Application of host DNA depletion kits in combination with in silico host read removal (e.g., map reads against reference-host genomes).	[16,24]
Inability to distinguish viable, VBNC, and dead cells	DNA from VBNC or dead cells may persist in the cheese matrix during ripening and lead to miscalculation and overestimation of actual abundance of the respective taxa.	Integration of shotgun metagenomics with viability-informed approaches (e.g., PMA treatment), acknowledging matrix-specific limitations. Additionally, apply multi-omics approaches including culturomics for validation of actually active taxa.	[19]
High heterogeneity across different cheese parts (e.g., core, rind, surface)	Distinct microenvironments (e.g., oxygen availability, salt, a_w_) create compartment-specific microbiomes, and analyzing only one compartment may miss capturing important taxa. Homogenization of the different compartments may introduce biases in microbiome composition.	Rind and core sampling should be conducted separately, with adequate replicate subsampling to capture variability, avoiding unnecessary over-mixing of distinct cheese compartments.	[13,20,21]
Lack of absolute quantification (CFU/g vs. relative abundance)	Microbial composition is usually expressed as relative abundance. Thus true microbial loads cannot be directly estimated, limiting ecological interpretations.	Application of absolute quantification techniques (e.g., spike-in controls, flow cytometry, qPCR) to calculate absolute microbial loads.	[22]

## 4. Bioinformatic Tools for Shotgun Metagenomics

Shotgun metagenomics have become an indispensable approach for analysis and deeper understanding of microbial ecology. Compared to amplicon sequencing, which provides narrower taxonomic resolution, shotgun metagenomics offer multi-level insights into microbial communities. Nevertheless, shotgun metagenomics generates large datasets that require substantial storage and computational capacity for downstream analysis. These challenges can be addressed by powerful computing systems, cloud-based analysis platforms, and optimized bioinformatic pipelines. Moreover, selecting the appropriate bioinformatic tools is critical and depends on the specific objectives and stage of the analysis. In cheese metagenomics, this involves a series of key methodological decisions, including the stringency of quality control and host DNA removal, the choice of taxonomic profiling strategy and desired resolution, and, critically, whether to rely on read-based approaches for rapid, broad characterization or to implement assembly-based analyses enabling genome reconstruction and functional context. Typical workflows for cheese shotgun metagenomics often follow defined steps including (i) quality control of raw sequencing data, (ii) taxonomic profiling, (iii) binning and MAGs reconstruction, and (iv) functional analysis [26]. Below, we discuss bioinformatic tools applied in cheese microbiome research. 

### 4.1. Quality Control: Ensuring Data Accuracy

The initial step in shotgun metagenomic analysis is the quality control of the raw sequencing reads. This procedure typically includes removing all artificial sequences introduced during library preparation for next-generation sequencing, such as trimming adapters. Additional QC steps include filtering out low-quality bases, duplicate read removal (to eliminate potential PCR artifacts), checking GC-content distributions, and removing host or contaminating DNA. Multiple tools have been employed for this purpose. FastQC “http://www.bioinformatics.babraham.ac.uk/projects/fastqc/ (accessed on 5 November 2025)” and MultiQC [27] are diagnostic tools, generating visual representations of read quality. For adapter and low-quality read trimming, commonly used tools include Cutadapt [28], TrimGalore [29], and Trimmomatic [30]. Host or contaminant genome removal is usually carried out with workflows such as KneadData “https://huttenhower.sph.harvard.edu/kneaddata (accessed on 5 November 2025)” which use read mappers like Bowtie2 [31] or the Burrows–Wheeler Aligner (BWA) [32]. Additionally, BEDTools [33] and SAMtools [34] are widely used to further support file format handling and filtering [20]. bcl2fastq performs demultiplexing and trimming of Illumina reads, while NanoPlot [35] is used to visualize long-read quality. More advanced tools, such as Fastp [36] and BBTools [37], combine quality check and trimming in a single step, offering simplified handling of large-scale datasets. Effective quality control is particularly important in cheese microbiome studies, as microbial DNA yields may be low. Host DNA contamination may also affect taxonomic and functional profiling leading to biased conclusions [16].

### 4.2. Taxonomic Profiling: Identifying the Participants in the Cheese Microbiome

Taxonomic assignment is a central step in shotgun metagenomics studies. There are multiple approaches for taxonomic characterization. Marker gene-based methods such as MetaPhlan3/MetaPhlan4 [38,39] allow species-level identification using clade-specific marker genes. For strain-level resolution, extensions such as StrainPhlAn [40] or PanPhlAn [41] are frequently employed. An alternative approach concerns k-mer taxonomic profiling, using Kraken2 [42], (coupled with Bracken [43] for better abundance resolution), which classifies reads by matching short DNA fragments (k-mers) against large reference databases. Other classifiers include Centrifuge, which uses compressed indexes based on the Burrows–Wheeler Transform (BWT) and the Ferragina–Manzini (FM) index, enabling efficient classification against large databases. Kaiju follows a different approach by translating sequencing reads into protein sequences and aligning them against reference protein databases such as NCBI nr, making it effective for identifying both microbial and viral taxa. Because microbial and viral genomes are rich in protein-coding genes, protein-level classification can improve taxonomic assignment in complex matrices such as cheese, as protein sequences are often more conserved at the functional level than the underlying nucleotide sequences [44]. All taxonomic profiling approaches described above are read-based and are typically applied for rapid community-level or strain-resolved surveys.

### 4.3. Assembly, Annotation, and Functional Assignment

Assembly and annotation are critical steps in shotgun metagenomics, turning raw reads into interpretable genetic information. Recent approaches employ MEGAHIT [45], metaSPAdes [46], or IDBA-UD [47] for efficient and accurate de novo assembly of contigs. The generated contigs can be annotated with MetaGeneMark [48], which predicts coding sequences, while gene abundance can be estimated by mapping reads back to the contigs with Bowtie2 [31]. Functional annotation can be performed by PROKKA [49] or HMMER/KOfam [50,51] to predict KEGG functionalities. Functional profiling tools have been developed to provide a more detailed insight into functional potential and to reconstruct or quantify metabolic pathways in complex ecosystems such as those found in cheese. Alignment- and Hidden Markov Model (HMM)-based approaches offer complementary perspectives by detecting conserved motifs and protein families beyond those represented in curated databases. DIAMOND [52] is a fast protein aligner that compares metagenomic reads or predicted proteins against large databases such as KEGG and SEED, supporting the identification of homologous proteins and the reconstruction of a functional profile. In contrast, HMMER, based on HMMs, recognizes conserved patterns in coding sequences and can detect distant homologs or functional sequences even if they do not fully match database entries. EggNOG-mapper [53] is a functional annotation tool based on orthologous groups (OGs). It supports multiple aligners (DIAMOND, MMseqs2 [54], HMMER) and allows ORF identification from assembled contigs via Prodigal [55]. Beyond gene functionality, EggNOG-mapper predicts KEGG, GO, EC, CAZy, and COG terms, as well as PFAM and SMART protein domains. It accepts both read-level and contig-level inputs and generates functional profiles, making it widely used in metagenomic studies. SUPERFOCUS [56] has also been employed for functional profiling. It is a framework that uses DIAMOND against the SEED database, allowing for multi-level hierarchical investigation of functions. GhostKOALA [57] is a web tool for direct protein assignment to KEGG Orthology (KO) groups, which can then be linked to KEGG pathways and hierarchical functions, supporting the reconstruction of metabolic maps. Read-based approaches can connect the different taxa to their metabolic pathways. The datasets derived from shotgun analysis can be further investigated for functional prediction by HUMAnN (HMP Unified Metabolic Analysis Network), which is one of the most used pipelines for detecting microbial pathways in whole ecosystems. HUMAnN 2/3 [58] initially maps reads to microbial pangenomes, thereby identifying functions, linking them to specific taxa, and generating abundance outputs of gene families. Subsequently, metabolic pathways can be reconstructed using MetaCyc [59], along with coverage and abundance calculations.

Of note, this stage of analysis relies on a central decision regarding whether to apply read-based approaches for rapid, broad profiling or assembly-based workflows to achieve strain-level resolution and gene-context analysis. Read-based methods prioritize speed and sample throughput, whereas assembly-based approaches emphasize genomic context, functional linkage, and higher resolution at the cost of increased computational demand. When required, combining both strategies can provide a comprehensive view of cheese microbiome structure and function.

### 4.4. Specialized Prediction Tools

BAGEL4 [60] is a web tool that identifies biosynthetic gene clusters of bacteriocins and ribosomally synthesized and post-translationally modified peptides (RiPPs). Input DNA sequences are translated and then scanned for conserved patterns and mapped against peptide databases. The predicted ORFs are annotated by BLAST+ 2.17.0 searches, and the generated clusters are visualized together with homologous peptide alignments, making BAGEL4 important for predicting antimicrobial potential. AntiSMASH [61] is a useful tool for mining secondary metabolite biosynthetic gene clusters (BGCs). It predicts cluster functions and potential metabolites from biosynthetic pathways identified in genomes and metagenomes. Even more specialized functional analyses include the annotation of carbohydrate-active enzymes (CAZymes) with dbCAN [62], a web tool that uses HMMs based on the CAZy database to investigate domain-level carbohydrate metabolism [63]. CarveMe [64] is an essential tool for generating genome-scale metabolic models, enabling the reconstruction of an organism’s full metabolism and its integration into community-level networks to predict total interactions and behaviors. Similarly, COBRApy [65] relies on metabolic models for flux balance and variability analyses and for simulating multiple gene essentiality tests. ARG detection can be performed with the AMR++ v3.0 pipeline, which operates against the MEGARes database and identifies antimicrobial resistance genes [66], while ShortBRED can also identify virulome profiles when screening reads against the appropriate database [67] (e.g., VFDB). Complementary approaches include RGI, ARG-ANNOT [68], ABRicate “https://github.com/tseemann/abricate (accessed on 5 November 2025)”, and the ResFinder [69]. CRISPR arrays (e.g., CRISPRCasFinder [70], CRISPRTarget [71]) can identify past phage exposure of microbiome taxa. CRISPRFinder is a specialized tool for detecting CRISPR-Cas loci, which are widely used to study microbial adaptive immunity and phage–host interactions. Virome recovery from contigs can also be achieved with VirSorter, VirSorter2 [72], and VIBRANT [73], while CheckV [74] is used to evaluate the completeness and quality of viral genomes. PHASTER [75] and Prophage Hunter [76] can further annotate prophages in MAGs. Additional information on the genetic context and transfer potential of genes can be obtained using plasmid detection tools such as PlasFlow [77], PlasmidFinder [78], Platon [79], and geNomad [80], as well as with IntegronFinder [81] and MobileElementFinder [82].

### 4.5. Mixed Pipelines

MEGAN (MEtaGenome ANalyzer) [83] enables taxonomic and functional annotation and visualization of metagenomic reads by mapping them to known protein databases (e.g., SEED, and KEGG), thus bridging the gap between taxonomic identity and metabolic potential. Taxonomy is assigned by aligning the reads with DIAMOND using the lowest common ancestor (LCA) algorithm, while mapping to KEGG or SEED hierarchies expands the analysis towards functional potential. MOCAT [84] and Parallel-Meta 3 [85] are two mixed toolkits that offer broad metagenomic data handling, including QC, taxonomic assignment, and functional prediction. The MOCAT pipeline integrates trimming and filtering of raw reads, mapping for removal, extraction or quantification of reads (e.g., host DNA), de novo assembly and quality assessment based on k-mers with SOAPdenovo [86], and mapping with BWA or SOAPaligner [87]. It also performs gene prediction using Prodigal or MetaGeneMark. Parallel-Meta 3, although originally based on 16S rRNA marker genes, can also process shotgun datasets. It extracts 16S fragments using Hidden Markov Models based on the SILVA database, aligns them with Bowtie2 against a curated database, and performs taxonomic assignment and phylogenetic analysis. Parallel-Meta 3 further offers statistical analyses and functional prediction using the PICRUSt [88] algorithm and KEGG pathways. Phylogenetic analysis of shotgun data can also be achieved through fetchMG [84], which extracts single-copy marker genes conserved across species for comparative genomics and phylogeny building, thereby avoiding biases from multi-copy or variable genes. SourceTracker is a Bayesian framework used to estimate the taxonomic contributions of different sources in metagenomic datasets, allowing the identification of contamination and microbial origin [89]. This tool has been applied to quantify the relative contributions of multiple microbial reservoirs, such as raw milk, whey, herd feed, teat skin, rennet, brine, and food-contact surfaces, to the formation of the final microbial composition of the raw-ewe-milk Idiazabal PDO cheese [11]. Overall, the multi-layered pipelines reinforce the interpretational potential of shotgun metagenomics providing global insights into the cheese microbiome and its underlying functional attributes.

### 4.6. MAGs: From Contigs to Genomes

For MAG recovery, binning tools such as CONCOCT [90], MetaBAT2 [91], and MaxBin2 [92] are often used to group contigs based on sequence composition and coverage, allowing the reconstruction of draft genomes from individual taxa from complex cheese communities. The quality and completeness of MAGs can then be evaluated with tools such as CheckM [93] or BUSCO [94], while their taxonomic profiling can be achieved through GTDB-Tk [95]. MetaWRAP [96] is a pipeline that combines multiple tools for shotgun metagenomics, supporting raw read processing, assembly, binning, and MAG construction. It also performs quality control, taxonomic analysis, and bin refinement, making it highly effective for improving MAG quality. Platforms such as anvi’o [97] and nf-core/mag [98] have also been used for retrieving KEGG functions from assemblies and MAGs. Additional phylogenetic information can be obtained by MAFFT [99], a multiple sequence alignment tool, and PhyML [100], which analyze the alignments to generate maximum-likelihood trees. The resulting phylogenies can be visualized with iTOL [101]. Tools such as BusyBee [102] are used for clustering/binning sequences based on composition and coverage, while RecruitPlotEasy [103] for visualization of reads against reference genomes to confirm populations at the strain level.

### 4.7. Statistics and Visualization

Multiple statistical and visualization packages have emerged to support all these outputs and enrich them with ecological meaning. The R programming language has been extensively used with multiple packages such as phyloseq [104], vegan, ComplexHeatmap [105], ade4 [106], pheatmap, and ape [107] for ordination, clustering, and diversity analyses. Biomarker detection can be achieved via LEfSe [108] and network visualization with Gephi-0.10.1 [109]. There are also web tools like MicrobiomeAnalyst [110] providing multiple statistical test and visualization options, or multivariate analysis software like SIMCA enabling microbial-functional connections. Other common visualization tools include Krona [111], Pavian [112], and GraPhlAn [113]. Table 2 summarizes the most commonly employed tools across key workflow steps, along with their applications, strengths, and limitations.

## 5. Shotgun Metagenomics for the Analysis of Cheese Microbiome

Research on cheese microbiota has advanced significantly through the application of metagenomic analyses. The existing literature demonstrates a great diversity of microbial roles, from fermentation agents to opportunistic contaminants and foodborne pathogens. A common theme across cheese microbiology is the symbiosis of domesticated starters and wild non-starter lactic acid bacteria (SLAB and NSLAB), driving the organoleptic and technological characteristics of cheese during fermentation and ripening.

### 5.1. SLAB Dominance Across Different Cheese Types and NSLAB Enrichment

SLAB including *Streptococcus thermophilus*, *Lactobacillus delbrueckii*, and *Lactococcus lactis* are domesticated species that have been used in cheese production for centuries. They are essential in early fermentation stages due to their central role in acidification, proteolysis/lipolysis, and the initiation of flavor formation. Shotgun metagenomics have been applied in multiple cheese types and results consistently show SLAB prevalence. Ruž’a and Wagashi cheeses were characterized by high abundances of the starter *Lc. lactis*, co-occurring with notable levels of *Lactococcus raffinolactis*, *Leuconostoc mesenteroides*, and *Streptococcus infantarius* [119]. Similarly, mountain Caciotta cheese exhibited *S. thermophilus* and *Lb. delbrueckii* as dominant taxa, and genomic strain-level diversity distinguished producers [114,123]. A comparative study of artisanal and industrial Greek Feta cheeses indicated that artisanal samples were dominated by *Lc. lactis*, while industrial Feta showed *S. thermophilus* and *Lb. delbrueckii* subsp. *bulgaricus* as the main starters [17]. Another metagenomic study on Greek artisanal cheeses (Sfela and its variants) found that while *S. thermophilus* and *Lc. lactis* were the main starter cultures in the industrial cheeses, in one artisanal variant, *Lc. lactis* notably appeared as NSLAB alongside *Tetragenococcus halophilus*. Consistent findings were reported for Argentine cow’s milk cheeses, dominated by members of *Enterococcus*, *Lactococcus*, and *Streptococcus* [124]. Natural whey starters of Swiss Gruyère cheese were dominated by *S. thermophilus*, *Lb. delbrueckii* subsp. *lactis*, and *Lactobacillus helveticus*, with the latter occurring as multiple co-existing strains [125]. Moreover, metagenomic mapping of different cheese types revealed thermophilic starters (*S. thermophilus*, *Lb. delbrueckii*) as predominant in mold-ripened cheeses, while *Lc. lactis* dominated soft smear cheeses, acting as primary acidifiers [126].

In contrast, wild NSLAB are being introduced into the cheese microbiome from raw materials, utensils, or the environment. After the initial acidification and as fermentation proceeds, NSLAB gradually increase with co-occurring metabolism events leading to texture and aroma formation. These taxa, with their broad metabolic potential, can thrive under the environmental shifts during ripening and survive under high-salinity, high acidity, and low-water-activity conditions. In aged Cheddar cheese, up to 32 months, *Lc. cremoris* dominated young cheese but declined as ripening progressed, while *Lcb. paracasei* became increasingly abundant [19]. Production practices largely affect SLAB/NSLAB balance, as well. Comparative investigations into Portuguese cheeses highlighted how the production process (PDO vs. non-PDO) influenced the microbial ecosystem characteristics and the prevalence of distinct taxa. More specifically, PDO cheeses showed greater microbial diversity, in *Leu. mesenteroides*, *Lc. lactis*, and *Lactiplantibacillus plantarum*, while non-PDO cheeses presented distinct profiles with higher prevalence of *Enterococcus durans* and *Lacticaseibacillus rhamnosus* [116]. In addition, the microbial composition and functional potential were found to be characteristic of Buffalo Mozzarella cheese from different regions. Overall, *S. thermophilus*, *Lb. helveticus*, *Lb. delbrueckii* ssp. *delbrueckii* were some of the core species, while *Lc. lactis*, *Enterococcus italicus*, *Liquorilactobacillus ghanensis,* and *Pediococcus parvulus* were discriminators of the samples from different regions. Distinct microbial patterns further contributed to region-specific volatile fingerprints are discussed below [14]. Such observations reveal how artisanal cheesemaking preserves NSLAB patterns shaping products with unique characteristics. Given their origin and ecological behavior, many NSLAB can also be considered adventitious microorganisms, acting as naturally occurring colonizers that link the internal cheese microbiota with environmental reservoirs. The co-existence of diverse microbial taxa, including both domesticated SLAB and environmentally adapted NSLAB, indicates the intricate microbial interactions that define cheese properties and organoleptic characteristics.

### 5.2. Environmental Reservoirs Shape Communities: Adventitious and Miscellaneous Microorganisms Beyond NSLAB

Processing environments serve as external sources of microorganisms, strongly influencing the formation of cheese microbial communities, and can be major determinants of cheese properties. Additional adventitious microorganisms, apart from NSLAB, are unintentionally introduced from the production environment or raw materials and contribute to the development of the cheese microbiota, both in the rind and the core, particularly in raw-milk and surface-ripened varieties. They can enter the cheese from brines, air, processing surfaces, or milk and they can survive under high-salinity, low-oxygen, or acidic environments due to stress-tolerance mechanisms and biofilm formation. Shotgun metagenomic analyses have revealed that these taxa could include both beneficial and problematic species going deeper than traditional culture-based analysis [20].

Typically, traditional culture-based techniques have a limited ability to detect “difficult-to-culture” microorganisms. As a result, they may provide inadequate information on microbial diversity, which can include stress-tolerant or slow-growing species that play a significant role in cheese ripening and properties [3]. Shotgun metagenomics has also offered new insights into this category of difficult-to-recover microorganisms. These “invaders” can display diverse roles in technological properties of the cheese such as enhancing lipolysis or development of pigment on the rind, while others may interfere with LAB, cause spoilage or carry antimicrobial resistance genes (ARGs). Halotolerant and biofilm-forming taxa, frequently colonize cheese rinds and surfaces, especially in artisanal or raw-milk cheese varieties [11,20,127].

Production process effects were evident in Idiazabal cheese, where factors such as the microbiology of feed, teat skin, brine, and rennet significantly influenced cheese microbial composition and technological potential. Shotgun metagenomics revealed that up to 41% cheese microbiota could be attributed mainly to rennet, and to a lesser extent to brine and contact surfaces. Raw-milk microbiota were strongly influenced by feed and teat skin. All cheeses were mostly dominated by LAB (e.g., *S. thermophilus* and *Lc. lactis*), while environmental sources resulted in additional taxa including *Clostridium*, *Bacillus*, *Staphylococcus*, *Corynebacterium*, *Brevibacterium*, *Brachybacterium*, *Dietzia*, *Micrococcus*, *Psychrobacter*, *Chromohalobacter*, *Halomonas*, and *Methylobacterium*. All the environmentally introduced taxa were linked to various functional attributes like biofilm formation and bacteriocin production, catabolism of amino acids and carbohydrates, and fatty-acid biosynthesis. Furthermore, *Staphylococcus-* and *Leuconostoc*-enriched samples contained more ARGs, while samples with rich staphylococcal presence displayed broader virulence factors. The study focuses on the importance of the cheese surroundings as reservoirs of functional and safety-related microorganisms that can enter the cheese microbiome [11]. Similar events were captured in the longitudinal metagenomic study of Alexa and colleagues on blue-veined Cabrales cheese, during which taxa of spoilage and safety concern have been observed [20]. Gram-negative, aerobic bacteria such as *Escherichia coli*, *Salmonella enterica*, *Acinetobacter*, and *Klebsiella pneumoniae* have been discovered in raw-milk and processing environments, which carried aminoglycoside, tetracycline, and β-lactam resistance genes. These bacteria have also been observed in the early fermentation stages, but as ripening progressed in the cave environments, they were displaced by cave-adapted genera such as *Tetragenococcus*, *Corynebacterium*, and *Brevibacterium*. This coincided with a significant reduction in ARG abundance, suggesting that microbial food adaptation contributes to a safer final product.

### 5.3. Rind Ecology: Halophiles/Halotolerants and Their Functions

Cheese architecture contains compartments with distinct microflora and characteristics. The strongly fermentative and proteolytic SLAB usually predominates in the core ecosystem, while a more “open” microbiological niche is observed in the rind, which can contain environmental taxa, yeasts, or stress-tolerant members. This differentiation is of particular interest and exploring each cheese domain separately may further explain the resulting microbiome and sensory attributes.

More specifically, rinds of cheeses are often characterized by dominant non-starter, adventitious taxa, including Gram-negative genera such as *Pseudoalteromonas* and *Psychrobacter*. Using an expanded dairy genome catalog to map rind metagenomes from two smear-ripened and one blue-veined PDO cheeses, Almeida and colleagues found these genera among the prevalent taxa and emphasized that they are not deliberately inoculated, but likely arise from brines, cheesemaking tools, and ripening environments [126]. Their high abundance suggests strong contributions to rind development and cheese characteristics rather than mere spoilage. Another metagenomic analysis has also revealed the presence of halophilic and halotolerant taxa obtained from cheese rinds. This study has shed light on the coexistence of Gram-positive and Gram-negative bacteria, across 13 artisanal cheeses. Regarding the Gram-negative taxa, *Halomonas*, *Psychrobacter*, *Pseudoalteromonas*, and *Vibrio casei*/*Vibrio litoralis* were mainly enriched in washed-rind cheeses. These microbes seem to increase in high-moisture cheeses, and their functional roles require further investigation [21]. Some adventitious taxa, including *Halomonas* and *Haloquadratum*, could even originate from artisanal handlings including the use of sea salt or aging environments. Multiple contigs recovered in the same study were found to carry genes that participate in amino acid and fatty-acid metabolism, likely connected to desirable flavor. Moreover, bacteriocins, mostly belonging to class II were also encoded across the identified contigs [23]. Rind-associated microorganisms have been often linked with the presence of various ARGs [12]. In addition, potentially transferable ARGs could be mapped on contigs belonging to *Enterobacteriales* (e.g., *Serratia*/*Providencia*) in Brazilian artisanal cheeses [21,127]. These observations reveal the key role of rind as an interface between the cheese and its environment, where the microbial composition and the specific biochemical processes taking place in this compartment contribute to the cheese’s distinctive character.

### 5.4. Yeasts as Adventitious Taxa in the Cheese Environment

Likewise, yeasts have a functional role as members of the microbial diversity and ecology of cheese. Shotgun metagenomics and ITS sequencing have revealed both dominant and accessory taxa across multiple studies. Yeasts including *Debaryomyces*, *Kluyveromyces*, *Pichia*, *Candida*, and *Rhodotorula* were found in Feta PDO cheese [17]. *Geotrichum candidum* has been identified as a main species in cheese rinds, causing preliminary deacidification and facilitating microbial succession by creating favorable conditions for the secondary bacterial and fungal communities [13]. *Debaryomyces hansenii*, a salt-tolerant yeast, has been observed as a dominant species in cheese surfaces and brine solutions in smear-ripened cheeses, contributing to proteolysis and aroma development [126,128]. In artisanal cheeses, the southern samples have been dominated by *D. hansenii*, while cheeses from southeastern regions, including Serro and Canastra, exhibited broader fungal diversity [119]. Metagenomic analysis revealed yeasts including *Diutina catenulata*, *Kodamaea ohmeri*, *Trichosporon* sp., and *Moniliella* sp., alongside *D. hansenii* [127]. Finally, *Kluyveromyces marxianus* was among the predominant species found in Wagashi cheese.

### 5.5. Technological and Ecological Drivers of Cheese Microbiome Structure

The structure of the cheese microbiome is shaped by multiple interdependent factors during maturation, including milk origin, heat treatment, pH and salinity, ripening time and environment, and production scale. Understanding the impact of these factors on cheese microbiome is crucial, as it not only concerns the technological and nutritional values of the product, but also defines its safety. Among these, milk treatment is of particular importance.

Raw-milk cheeses exhibit greater microbial diversity and complexity compared to pasteurized counterparts due to naturally occurring microbes in raw milk that persist through processing [129,130]. Due to the lack of pasteurization, high diversity stems from the naturally occurring microbes present in milk, which remain preserved during processing and may contain beneficial microorganisms. Fontana and colleagues conducted a broad survey of 128 Italian raw-milk cheeses and demonstrated that microbial composition is more strongly associated with the production site and natural whey cultures rather than with the Protected Designation of Origin (PDO) label [22]. Dominant taxa included *Lb. helveticus*, *S. thermophilus*, *Lc. lactis*, and *Leuconostoc* species, each displaying distinct enzymatic repertoires that affected the organoleptic qualities and bioactive potential of the final products. Functional gene profiling revealed that each cheesemaking site harbored a distinct enzymatic repertoire, encompassing pathways for vitamin, glutathione, and bioactive compound biosynthesis, emphasizing that microbial diversity contributes to both sensory uniqueness and nutritional benefits.

Pasteurized-milk cheeses, in contrast, exhibit lower microbial diversity and complexity, mainly due to the elimination of most indigenous microorganisms. Heat treatment allows the starter cultures (e.g., *S. thermophilus* and *Lc. lactis*) to thrive and dominate fermentation, leading to a predicted and consistent microbial and sensory profile. De Sant’Anna et al. and colleagues observed that pasteurized-milk cheeses demonstrate limited microbial diversity and resistome, with residual ARGs mainly associated with *Bacillus cereus* [12]. These findings support that pasteurization leads to a more predictive outcome in both microbial and gene complexity and a decreased potential for resistance gene dissemination, ensuring greater process control, albeit at the expense of microbial and functional diversity in the final product. Cheesemaking environments also act as crucial microbial determinants. A study using metagenomics revealed that artisanal processing environment in cheese fermentation may be a dominant determinant for the formation of microbial populations and complexity [131]. Sampling from 137 cheese rinds across ten countries revealed that rind type (washed, bloomy, natural) and the surface moisture can be strongly linked to microbial diversity while other factors like location, milk source, or pasteurization status had less influence. In vitro analyses demonstrated that artisanal manipulations such as brine washing, control of humidity, and pH selectively favor specific microbial taxa such as *Brevibacterium*, *Corynebacterium*, *Staphylococcus*, and *Penicillium*. These findings demonstrate that the cheese processing practice can reproducibly shape microbial ecosystems, independent of geographic origin. Technological interventions, as evident, have a great influence on the cheese microbial evolution.

A study comparing Mediterranean artisanal cheeses from Portugal, Spain, Italy, and Morocco showed that LAB were commonly found across all cheeses, with *Lactococcus*, *Streptococcus*, and *Lactobacillus* being the prominent genera in the Portuguese and Spanish cheeses. The Italian Squacquerone cheese was mainly dominated by *S. thermophilus*. Moroccan Jben cheeses contained significant numbers of *Enterococcus*, *Klebsiella*, *Escherichia*, and *Citrobacter*. These cheeses also exhibited the highest abundances of ARGs and virulence factors, features characteristic for spontaneously fermented raw-milk cheeses [132]. These results highlight that spontaneous fermentation of raw milk under variable hygiene conditions can increase both microbial and functional diversity, though sometimes at the expense of safety.

Overall, technological parameters are crucial determinants of cheese ecology. The impact of production methods and the processing environment on the cheese microbiome and its functionality can be assessed in detail by shotgun metagenomics, both qualitatively and quantitatively. Notably, this approach not only captures compositional differences but also links them to dynamic biological processes, such as microbial competition, stress adaptation, and horizontal gene transfer, that remain inaccessible to conventional microbiological techniques. This integrated evaluation of microbial functions, together with the technological and environmental factors that shape them, represents a core component of contemporary dairy science.

### 5.6. Microbial Succession During Ripening

**Figure 1:**
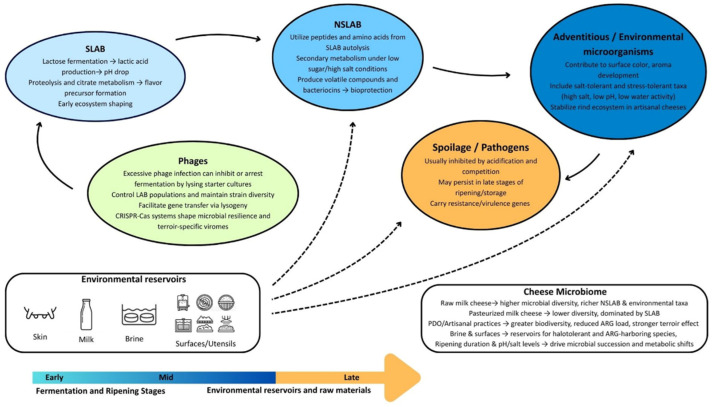
Conceptual diagram illustrating the dynamic relationships among the main microbial groups shaping cheese microbiomes during fermentation and ripening.

The cheese microbial community undergoes continuous transitions during production that may be influenced by multiple factors related to the manufacturing methods applied. The overall identity of cheese is strongly defined by the co-existing microbiota and their interaction within the product’s matrix. Shotgun metagenomic comparison of raw and pasteurized cheeses showed differences in microbial dynamics. During the first month of ripening, the core of the pasteurized samples was dominated by *S. thermophilus*, while during the second month *B. cereus* also appeared. The rind was initially dominated by *S. thermophilus*, but during the second month a marked increase was observed in salt-tolerant, Gram-negative taxa such as *Halomonas hibernica*, *Halomonas alkaliphila*, *Pseudoalteromonas prydzensis*, *Psychrobacter* sp., *V. casei*, and *V. litoralis*. The core of unpasteurized cheese A during the first stage of ripening was dominated by *S. thermophilus*, *Lcb. paracasei*, *Lc. lactis*, *Lb. delbrueckii*, and *Lb. helveticus*. By the second month, *Lcb. paracasei* and *Lc. lactis* increased, with the latter showing a subsequent decline. The rind of the same cheese was also dominated by LAB early on, but as ripening progressed, *Staphylococcus equorum* and *Halomonas* spp. became more abundant. In unpasteurized cheese B, the core showed a similar composition in the first month, with significant abundances of *S. thermophilus*, *Lb. helveticus*, *Lc. lactis*, and *Leuconostoc pseudomesenteroides*. Following, during the second month, there was an increase in *Halomonas* and *Psychrobacter* species. On the rind, *Staphylococcus* showed high abundance early, while in later stages there was a co-occurrence of halotolerant taxa. Reconstruction of MAGs identified *S. thermophilus* as a core member throughout ripening [12]. Shotgun metagenomics applied to Austrian artisanal Vorarlberger Bergkäse cheese revealed clear microbial and metabolic transitions during ripening. Samples at 30 d of ripening exhibited degradation of residual lactose, lactate, and citrate mostly attributed to *Staphylococcus* species. At a later stage (i.e., 90 d) the degradation of small molecules such as free amino acids and fatty acids took place, which was linked to the presence of *Brevibacterium* and *Corynebacterium* [133]. These findings highlight the strong influence of the ripening environment on microbial succession, following a typical pattern in which starter-associated taxa dominate the early stages of ripening, while progressive physicochemical changes such as lactose depletion, pH reduction, and decreased water activity drive temporal shifts in microbial community composition as maturation proceeds [134]. The stages of microbial succession and the associated ecological interactions are presented in Figure 1.

### 5.7. Spoilage Microbiota and Food Safety Considerations

Shotgun metagenomic analysis of surface-ripened cheeses detected *Brochothrix thermosphacta*, a known dairy spoilage-associated bacterium, as part of the cheese microbiome [135]. This investigation also showed that *L. monocytogenes* abundance varied across samples and was positively correlated with high water activity and *S. thermophilus*, while showing negative associations with *Brevibacterium* and *Lactococcus* species. No direct relationship was observed within *B. thermosphacta* and *L. monocytogenes* growth. Although *L. monocytogenes* growth differed significantly among cheeses, it was consistently lower in raw-milk cheeses compared with pasteurized-milk cheeses. Additional pathogens such as *Staphylococcus aureus* and *L. monocytogenes* were detected in Feta, their levels related to low abundances [17].

A major theme in the literature is the spoilage risk, and it has been addressed in several papers. *Thermus* species are considered to cause pink discoloration defects in cheese and are linked to carotenoid production, and, in particular, lycopene [136]. In Gouda samples with defects related to cracks, shotgun metagenomics enabled the reconstruction of *T. halophilus* and *Loigolactobacillus rennini* MAGs. Further functional prediction showed that *Loil. rennini* contained genes related to the production of GABA, putrescine from ornithine, and cadaverine from lysine catabolism linked to the observed quality defects and spoilage through amine accumulation, indicating that the identified amine-producing pathways may underlie the observed spoilage associated with surface defects [10].

Shotgun metagenomics, used for the analysis of raw-ewe-milk Idiazabal cheese and various environmental factors, revealed that undesirable bacteria, including *Staphylococcus* and *Pseudomonas*, were highly abundant in whey, suggesting their dissemination from cheese. The presence of *Pseudomonas* in both whey and cheese could be attributed to its high abundance in rennet. Pathogenicity and antimicrobial factors linked to taxa such as *Psychrobacter*, *Lactococcus*, *Staphylococcus,* and *Leuconostoc* were detected across all samples, with cheese, whey, grass, and commercial feed harboring the highest loads. Two of the most frequently identified virulence factors (VFs) contained prophage and nisin resistance genes, underscoring the potential risks in the microbiome of both cheese and the environment [11]. Brine may also act as drives of the cheese ecosystem. For example, spoilage taxa have also been discovered during metagenomic analysis on the cheese brines studied. Results obtained showed that cheese brine acts as an active microbial reservoir that is dominated by halophilic taxa such as *T. halophilus*, *Chromohalobacter*, and *Halanaerobium*, alongside *Loil. rennini* and *Staph. equorum*. These species are associated with off-flavors and surface defects in washed-rind cheeses. The existence of such taxa underscores the importance of hygienic management related to brine, in order to prevent spoilage events and ensure product safety [128]. The main microbial groups detected by shotgun metagenomics across the different cheese types are summarized in Table 3.

## 6. Functional Insights from Shotgun Metagenomics of Cheese

As already mentioned, shotgun metagenomics has expanded the analytical field towards functional predictions, including gene profiling and pathway reconstructions of different cheeses. The studies selected of this review capture a wide range of functional analysis possibilities such as phage–host interactions and CRISPR/anti-CRISPR detection, correlation of specific taxa to sensory attributes, bacteriocins production, and resistome potential.

### 6.1. Metabolic Pathways and Sensory Attributes

In Caciotta cheese, pathways connected to amino acid, citrate, and lipid metabolism were detected through in silico functional analysis and were associated with VOCs such as acetic acid, 2-butanol, and acetoin. Typicity related to the different producers was defined by the distinct microbiomes and VOCs composition, reflecting unique metabolic potential [114,123]. In Buffalo Mozzarella, functional analysis revealed that while pathways for sulfur, amino acid, lipid, and vitamin metabolism were more complete in cheeses from the Caserta province, cheeses from Salerno showed a more diverse volatile profile, with ethyl acetate, butanoic acid, and diacetyl being enriched. Taxa–VOC correlation networks showed consistent associations of *Lc. lactis* in Caserta cheeses with butanoic acid and diacetyl, while in Salerno samples *Liql. ghanensis* and *P. parvulus* were linked with ethyl acetate and hexanal. These findings supported the impact of the terroir on the cheese microbiome and its consequent role in shaping distinct metabolic and volatile profiles [14]. In a metagenomics study of Brazilian cheeses, the milk adaptation module lacSZ locus was detected in *S. infantarius*, indicating probable horizontal gene transfer from *S. thermophilus* [78]. In another investigation, Elcheninov and colleagues explored fermented milk products through shotgun metagenomics [63]. The presence of secondary metabolite gene clusters was also observed. Suluguni-like, cottage, and bryndza cheeses contained broad CAZyme profiles, and furan biosynthesis genes were detected in all three, while suluguni-like and cottage cheeses additionally encoded genes of the aryl-polyene PKS cluster. Furan and furan-related compounds are odor-active volatiles. At low levels, they can influence cheese aroma and overall sensory balance contributing to flavor complexity, whereas higher concentrations may lead to off-flavors [63,137]. Ripening influence was determined in aged Cheddar cheeses. As ripening progressed, functions connected to DNA repair/acid resistance (*uvrA*), trehalose/osmoprotection operons, peptidases (pip), amino-sugar transport/degradation (*hisA*, *trpAC*), and ribosome-rescue response (*hflX*) started to dominate the cheese matrix. After propidium monoazide (PMA) application, viable cells were predominantly assigned to *Lcb. paracasei*, while *Lc. cremoris* signals notably dropped. The aforementioned functions were attributed to both taxa signals, thus suggesting compliance with ripening-driven dynamics [19].

### 6.2. Resistome and Mobile Genetic Elements

LAB are not widely connected with notable ARG loads compared to Gram-negative bacteria, although these loads can be affected by the product matrix and the production processes which follow. Irish cheeses harbored 40 antimicrobial resistance genes (ARGs) associated with lincosamide, multidrug, and fosfomycin resistance, among others, which were linked to 35 MAGs, while an additional 74 ARGs were identified on plasmids. None of the plasmid-associated ARGs were related to LAB [120]. Tan and colleagues showed that among various ready-to-eat fermented foods, dairy products, including cheese, exhibited the lowest diversity of ARGs [117]. MAG analysis related *S. thermophilus* to tetracycline and β-lactam tolerance, while genes containing determinants linked to the latter were also detected in *Lc. lactis*/*cremoris*. In the study of De Sant’Anna, resistome profiling demonstrated that ARGs were mainly connected with Gram-negative and staphylococcal rind communities rather than core LAB. *S. thermophilus*, which dominated early cores in pasteurized cheese and carried macrolide-resistant genes (*mls23S*), while the ARGs *fosB* (fosfomycin) and *bclL* (β-lactam resistance) appearing during the second month correlated with the presence of *B. cereus*. The rind community, during the same month, exhibited aminoglycoside (*a16S*), cationic peptide (*cap16S*), and macrolide (*mls23S*) resistance factors, corresponding mainly to the rise in *Halomonas* and *Vibrio* members. The macrolide resistance genes (*mls23S*) detected early in the core of unpasteurized samples were followed by increase in multidrug efflux pump gene *lmrD* and the rifampin resistance gene *rpoB* as ripening progressed. The increase in *Staphylococcus* and *Halomonas* species in the rind from the same samples, during the second month of ripening, was associated with the simultaneous appearance of quaternary ammonium resistance genes (*qacG*, *qacJ*) and metal resistance genes (*cadD*, *arsM*, *arsC*). By the second month, in unpasteurized cheese B cores, the appearance of aminoglycoside resistance markers (rrs and a16S) was linked to the parallel increase in *Halomonas*, *Psychrobacter*, and *Corynebacterium* species. On the rind, *qacG* co-occurred early with *Staphylococcus*, while at later stages, metal resistance genes appeared with the rise in halotolerant taxa. Reconstruction of MAGs identified *S. thermophilus* as a core member throughout ripening. Overall, these results suggest that ripening-related microbial shifts also determine the resistome dynamics [12]. A multi-factor investigation supported that production could act as significant reservoirs of ARG appearing in cheese. More precisely, analysis of 1,780 samples from 113 facilities indicated that over 70% of ARG genes were detected throughout multiple stages of production lines (e.g., raw materials, processing surfaces, and cheese), while approximately 40% of them were linked to mobile genetic elements (MGEs). Surfaces related to raw materials were mostly enriched in β-lactam/macrolide/aminoglycoside/tetracycline resistance genes. Further analysis connected genes related to tetracycline resistance to *Lc. lactis*, *Enterococcus faecalis*, *Enterococcus faecium*, and *Streptococcus parauberis*, macrolide resistance to *Lpl. plantarum,* and aminoglycoside resistance to *Staph. aureus*. Final cheeses shared a common core of ARG variants with samples from ripening and packaging surfaces. This observation may imply that dairy plant surfaces are a key factor influencing the resistome of cheese, thus a broad surveillance of the environmental factors is required [115]. Similarly, comparative shotgun analysis of probiotics, starters, and cheeses identified significant amounts of ARGs on MGEs, while the highest amount and diversity was detected in raw-milk cheeses without starters. Most contigs carrying, ARG related to disinfectant/tetracycline/macrolide/aminoglycoside/MLS/trimethoprim resistance, belonged mainly to *E. faecium* and *Lc. lactis*. Gram-negative taxa, containing potentially pathogenic species linked to raw-milk cheeses, also contained significant amounts of β-lactam and quinolone resistance genes. These findings focus on the importance of ARGs in microorganisms present in raw-milk cheeses compared to commercial starters [138]. Furthermore, comparative analysis of Portuguese cheeses showed that, despite overall compliance with European Commission microbiological safety standards, non-PDO cheeses exhibited a higher number and broader diversity of virulence and antimicrobial resistance associated genes than PDO cheeses, as evidenced by shotgun metagenomic profiling. In particular, virulence genes such as senB, ipfA, and aur were detected exclusively in non-PDO samples, while non-PDO cheeses also displayed a wider repertoire of resistance determinants, including genes associated with tetracycline, macrolide, and multidrug efflux or biocide resistance, whereas PDO cheeses consistently harbored fewer such genes overall. *Enterobacteriaceae* (e.g., *E. coli*/*Klebsiella*), staphylococci and enterococci were the most frequently ARG-associated taxa. Shotgun metagenomics further revealed extended-spectrum β-lactamase (*ESBL*) genes (e.g., blaTEM) and virulence factors (e.g., yfeA/B, iucA/D) associated with some of the aforementioned taxa [116]. A study investigating Brazilian artisanal cheeses, showed that tetracycline resistance genes (*tetK* and *tetS*) were mapped to rind staphylococci and core streptococci (including *S. thermophilus*), and quinolone resistance gene (*qnrD1*) with *Serratia*/*Providencia* was found in rind [63]. Finally, ARGs were enriched in bryndza samples, probably due to their rich microbial diversity compared to other cheese types analyzed in that study.

### 6.3. Bacteriocins

In the mining of previously published Cotija cheese metagenomics data, two previously unreported class II bacteriocins (QC1, QC2) were detected and classified as lactococcin-family peptides [122]. Similarly, shotgun metagenomics of cow’s milk artisanal cheeses from Argentina revealed additional genes related to class II bacteriocins, such as lactococcins, enterocins, and streptococcal bacteriocins in samples enriched in *Enterococcus* and *Streptococcus*. Further research identified a bacteriocin carrier as *E. faecium CRL1879*, whose antilisterial activity was confirmed in experimentally contaminated cheeses. These results shed light on the dual role of LAB members as contributors to both fermentation and bioprotection within the cheese matrix. In another study, lanthipeptide and other RiPP-like bacteriocin gene clusters were detected across all cheese metagenomes analyzed [63]. Suluguni-like cheese showed the highest diversity of RiPP-related genes, including nisin-associated clusters.

These findings highlight the potential of metagenomics to directly identify novel natural antimicrobial compounds through in silico analysis within the members of the cheese microbiome that would remain mostly undetected using conventional culture-dependent or targeted molecular approaches. However, in silico prediction cannot support the actual expression of the bacteriocin-associated genes. Additional experimental approaches are needed to verify actual antimicrobial activity.

### 6.4. Phage–Host Ecology, CRISPR, and Anti-CRISPR

Phage pressure is a real risk in dairy technologies, acting as an ecological regulator capable of collapsing dominant SLAB populations. Shotgun metagenomics in Gruyère natural whey starters shed light on the presence of multiple phages along with an *S. thermophilus* prophage. Moreover, it was demonstrated that *S. thermophilus* and *Lb. delbrueckii* contained a CRISPR-Cas defensive system, suggesting co-evolutionary dynamics between phages and their bacterial hosts in natural whey starters [125]. Thus, CRISPR-based monitoring paired with rotation or usage of mixtures of starters may help prevent fermentation failures due to phage outbreaks in the cheese matrix. Studies on Caciotta cheese support the presence of multiple phages associated with starter cultures, such as *Streptococcus* phage TP778L, *Streptococcus* virus DT1, phiAbc2m, *Lactococcus* phage bIL310, and *Lactobacillus* phage A2. The biodiversity and abundance variations in the detected phages were characteristic for the different producers and thus these viral communities could serve as process- and origin-specific biomarkers. Therefore, beyond the potential hazard caused, phages can also shape unique fingerprints of the same producer-/origin-specific cheeses [114,123]. Metagenomic analysis of both virome and microbiome of Brazilian Canastra cheeses revealed more than 1200 viral operational taxonomic units (vOTUs), belonging mostly to Caudovirales (particularly *Siphoviridae)*. About 28% of these viral entities were producer-specific, proving that specific phage communities come from distinct environments, representing artisanal practices. More specifically, 16 MAGS, with some of them belonging to *Streptococcus salivarius*, *E. coli*, *S. infantarius*, and *Streptococcus agalactiae,* contained various anti-phage genes (CRISPR–Cas), mostly containing spacers matching Caudovirales families. During the investigation, a putative novel *Streptococcus* 987-group phage and *Staphylococcus* phages (Rosenblumvirus) were also identified, which have a potential role in biocontrol applications [139]. Meta-analysis of various Irish cheese microbiomes discovered significant abundances of viruses, including *Siphoviridae* phages (e.g., *Lactococcus* and *Streptococcus* phages). Further analysis revealed a non-linear association between *Siphoviridae* and *Streptococcaceae*, showing positive correlations below and negative correlations above 15% abundance of the viral strain. CRISPR-based analysis highlighted once more the co-evolution of hosts and phages within the cheese matrix, revealing both anti-phage defense and anti-CRISPR proteins. Anti-CRISPR genes carried by *S. thermophilus* phages, with *AcrIIA6* being the most abundant, were observed in more than half of the samples, while CRISPRs of *Lactococcus* and *Staphylococcus* MAGs contained spacers of *Streptococcus* and *Lactococcus* phages [120]. Research on raw and pasteurized caprine milk cheeses identified the heat-resistant *Lactococcus* phage P680 in all analyzed samples, raising potential concerns since *Lc. lactis* may play a central role in the fermentation of these cheeses [121].

These studies highlight the value of metagenomic phage surveillance and CRISPR-based monitoring in guiding informed starter culture rotation and optimized mixed-starter strategies, thereby reducing the risk of fermentation failures. Moreover, understanding the balance between bacterial CRISPR-based defenses and phage anti-CRISPR mechanisms may help predict starter robustness and enhance consistency in cheese manufacturing.

## 7. Interpretive Limitations of Shotgun Metagenomics

Although shotgun metagenomics is a powerful tool for revealing “who is there” and “what genes are there”, it still has a number of limitations. Microbial data from the cheese matrix can be distorted due to host genome signal interference. Moreover, annotation of short reads may lead to insufficient characterization of ARGs loci (plasmid or chromosomal) and is unable to confirm their activity. Thus, additional long-read sequencing and targeted culture-dependent analyses may be needed to precisely map genes [12]. Capturing the exact active cheese microbiota at specific time points cannot be fully achieved through shotgun sequencing, as the extracted DNA can derive from live, dead, or VBNC cells. As a result, functional analysis is restricted to the predictive level, indicating potential rather than actual activity. Multi-omics analyses, combining metagenomics with transcriptomics, proteomics, and culturomics, can be very useful in clarifying both the presence and functionality, allowing for a more realistic characterization of the active microbiome [18,115]. Beyond these, the activity of phages and CRISPRs cannot be determined solely by shotgun metagenomics [114]. Finally, differences in wet-lab techniques lead to the generation of data that may need customized workflows and approaches. Currently, there is no universal bioinformatic pipeline applicable to every food metagenomics dataset and using different computational workflows may generate variable outcomes. In order to ensure reproducibility and consistency of the data presented and analyzed across studies, benchmarking of sampling, sequencing, and the application of downstream bioinformatics tools is required [132].

## 8. Future Perspectives

In next-generation sequencing-based food microbiome research, multiple advanced approaches, including long-read sequencing, metagenome-assembled genome (MAG) reconstruction, and integrated multi-omics frameworks, are increasingly being implemented. These developments are already enabling more precise and mechanistic links between production practices and microbiome structure, thereby facilitating the attribution of specific volatile fingerprints, metabolic pathways, and key quality determinants to defined microbial consortia [3,13,14,140]. Future perspectives should be focused on merging multi-omics approaches to bridge the gap between functional expression and taxonomic data of cheese microbiomes. The combination of shotgun metagenomics along with metatranscriptomics, metabolomics, and culture-based assays can provide a complementary view of the complexity of microbial interactions [3,141]. Innovative sequencing strategies for the absolute, viability-informed quantification of microbial taxa will provide improved ecological and functional accuracy, thereby enabling deeper insight into complex cheese ecosystems. Coupling real-time or near-real-time microbial monitoring systems with prediction of their functionality using modeling tools could be a promising way to improve production and enhance quality assurance regarding the distinctive organoleptic properties and safety risks of each different type of cheese [142]. Beyond multi-omics integration, forthcoming applications such as the design of synthetic microbiomes will evolve metagenomics from a descriptive tool into a predictive and engineering platform for precision cheese fermentation [143]. Such developments, combined with novel artificial intelligence (AI) bioinformatics tools, will make it possible to follow microbial changes, monitor fermentation progress, detect early signs of spoilage, and assess the presence of foodborne pathogens directly in the dairy plant [144]. In this context, digital twin technologies enable the creation of virtual replicas of cheese production that incorporate real processing parameters with in silico data enabling the precise simulation of microbial succession, metabolic outputs, and alternative production scenarios [145,146].

Moreover, the cheese microbiome has been suggested to serve as a vector for modulating the human gut microbiota [147]. Cheeses may act as a tool for microbiome engineering, delivering psychobiotic and immunomodulatory microorganisms as well as bioactive metabolites capable of shaping gut microbial structure and function [63,140].

## 9. Conclusions

Shotgun metagenomics has revolutionized cheese microbiology, moving the field beyond the classical recording of taxonomic inventories toward mechanistic insights into ecosystem-level interactions. This approach elucidates how raw materials, cheesemaking practices, and processing environments contribute to microbiome composition and product quality. Today, shotgun metagenomics provides the most comprehensive view of microbiome composition relative to other culture-independent molecular methods. Moreover, reconstruction of MAGs adds to detailed taxonomic resolution while enabling the detection of key microbial metabolic pathways, which link specific members of the microbiome to cheese technological and functional attributes. Through shotgun metagenomics, safety-related features such as ARGs and VFs can also be characterized alongside the monitoring of phage–host interactions and CRISPR–Cas systems, which are critical for starter culture robustness and process stability. When combined with other omics technologies, predicted pathways (e.g., proteolysis, lipolysis, amino acid catabolism, citrate metabolism) can be associated with quality attributes of cheese such as flavor development. Furthermore, shotgun metagenomics facilitates monitoring microbiome dynamics at critical production stages that may support the optimization of LAB and NSLAB communities to enhance safety and desirable technological and functional properties. Despite these advantages, variability in wet-lab protocols and downstream analytical approaches still limits consistent cross-study comparisons. Therefore, benchmarking sampling, DNA extraction protocols, and bioinformatics workflows are required. Additionally, shotgun metagenomics predicts functional potential, rather than revealing actual metabolic activity or gene expression. Its full potential can be achieved through integration with multi-omics approaches and experimental evidence, providing context-specific validation that connects genetics with phenotypic outcomes. Ultimately, all these advances lay the foundation for more resilient, safety-oriented, and quality-driven production of both industrial and artisanal cheese.

## Figures and Tables

**Table 2:** Bioinformatics tools most commonly employed in shotgun metagenomics for cheese.

Workflow Step	Bioinformatic Tools	Common Application in Cheese-Focused Studies	Strengths	Limitations	References
Quality control and removal of host DNA	FastQC, MultiQC, fastp, Cutadapt/TrimGalore/Trimmomatic, KneadData (with Bowtie2/BWA)	Quality control of raw sequencing reads, trimming of adapters and low-quality bases, removal of host-derived reads	Widely used, fast, and easily integrated into reproducible pipelines. Well-established preprocessing step in cheese metagenomics studies	Excessive trimming or filtering can lead to reduction in effective coverage. Dependent on accurate host reference genomes	[11,19,114,115,116,117,118]
Read-based taxonomic profiling	MetaPhlAn3/4, Kraken2 (coupled with Bracken), Kaiju	Identification of cheese microbial taxa and estimation of their relative abundance	Fast, assembly-free methods. MetaPhlAn supports robust species-level resolution. Kraken2/Bracken provides high sensitivity and improved abundance estimation. Kaiju enhances viral identification	Strongly dependent on the database used, thus exhibiting limited performance for poorly represented or novel taxa. Provide only relative abundances	[11,12,14,25,63,114,118,119,120]
Assembly and MAGs recovery	MEGAHIT, metaSPAdes, CONCOCT, MetaBAT2, MaxBin2, MetaWRAP, CheckM, GTDB-Tk	Assembly of metagenomic contigs and recovery of MAGs to explore microbial taxa and their functional potential	Enables recovery of high-quality genomes, identification of novel or low-abundance taxa. Provides improved functional analysis	Requires high sequencing depth and substantial computational resources, sensitivity to uneven coverage and contamination, MAG recovery favors genomes with high coverage and completeness	[10,12,14,20,63]
Functional profiling	HUMAnN, DIAMOND (SEED/KEGG), eggNOG-mapper, GhostKOALA	Identification of genes and metabolic pathways in cheese microbiomes, including functions involved in flavor development, proteolysis and lipolysis, vitamin biosynthesis	Enable comprehensive functional characterization of cheese ecosystems. Functions can be linked to specific taxa (HUMAnN), orthology-based tools (e.g., eggNOG-mapper) can provide broad functional annotation across multiple databases	Strongly dependent on reference databases and homology thresholds, predictive functional potential and not actual metabolic activity	[20,22,23,114,115,120,121]
Specialized functional prediction	BAGEL4, antiSMASH, dbCAN, ABRicate, CRISPRCasFinder	Detection of bacteriocin and RiPP clusters, secondary metabolite BGCs, CAZymes, ARGs, virulence-associated factors, CRISPR -Cas systems, and phages	Reveal technological potential, safety-related traits, and phage–host interactions relevant to cheese quality and starter culture stability	Represent predictive functional potential rather than actual expression or activity	[63,118,119,122]

**Table 3:** Microorganisms detected by shotgun metagenomics across different cheese types.

Microbial Group	Key Taxa	References
SLAB		
	*S. thermophilus*, *Lc. lactis*, *Lc. cremoris*, *Lb. delbrueckii* (subsp. *bulgaricus*/*lactis*), *Lb. helveticus*	[11,12,14,17,18,19,22,114,116,119,125,126,132]
NSLAB		
	*Lcb. paracasei*, *Lcb. rhamnosus*, *Lpl. plantarum*, *Leu. mesenteroides*, *Leu. pseudomesenteroides*, *Lc. raffinolactis*, *P. parvulus*, *Liql. ghanensis*, *E. durans*, *E. italicus*, *E. faecium*, *T. halophilus*	[12,14,18,19,116,119,124]
Yeasts		
Ascomycota	*D. hansenii*, *K. marxianus*, *G. candidum*, *Ko. ohmeri*, *Di. catenulata*, *Pichia*, *Candida*, *Rhodotorula*, *Moniliella*	[13,17,118,119,126,127]
Basidiomycota	*Trichosporon*	[127]
Adventitious taxa		
Actinomycetota	*Corynebacterium*, *Brevibacterium*, *Brachybacterium*	[11,12,20,133]
Bacillota	*Staph. equorum*, *Clostridium*	[11,12]
Pseudomonadota	*H. hibernica*, *H. alkaliphila*, *V. casei*, *V. litoralis*, *Psychrobacter*, *Pseudoalteromonas*, *Chromohalobacter*, *Acinetobacter*	[11,12,20,21]
Spoilage		
Bacillota	*Loil. rennini*, *Br. thermosphacta*	[10,135]
Deinococcota	*Thermus*	[136]
Pathogens		
Bacillota	*Staph. aureus*, *L. monocytogenes*, *B. cereus*	[12,17]
Pseudomonadota	*E. coli*, *Sal. enterica*, *Kl. pneumoniae*	[20,117]

## Data Availability

No data was generated during this study.

## Abbreviations

The following abbreviations are used in this manuscript:

Abbreviation	Meaning
AI	Artificial intelligence
ANI	Average Nucleotide Identity
ARG	Antimicrobial resistance gene
BGC	Biosynthetic gene cluster
BUSCO	Benchmarking Universal Single-Copy Orthologs
CAZyme	Carbohydrate-active enzyme
COG	Cluster of Orthologous Groups
CRISPR	Clustered Regularly Interspaced Short Palindromic Repeats
EC	Enzyme Commission Number
GTDB-Tk	Genome Taxonomy Database Toolkit
GO	Gene Ontology
HMM	Hidden Markov Model
HMMER	Hidden Markov Model Software for Sequence Analysis
HMP	Human Microbiome Project
KEGG	Kyoto Encyclopedia of Genes and Genomes
KO	KEGG Orthology
LAB	Lactic acid bacteria
LCA	Lowest common ancestor
LEfSe	Linear Discriminant Analysis Effect Size
MAG	Metagenome-assembled genome
NGS	Next-generation sequencing
NSLAB	Non-starter lactic acid bacteria
OTU	Operational taxonomic unit
PDO	Protected Designation of Origin
PKS	Polyketide Synthase
QC	Quality control
R	Statistical Programming Language
RiPP	Ribosomally synthesized and post-translationally modified peptide
R-M	Restriction–Modification System
SIMCA	Soft Independent Modeling of Class Analogy
